# Ubiquitin signalling in neurodegeneration: mechanisms and therapeutic opportunities

**DOI:** 10.1038/s41418-020-00706-7

**Published:** 2021-01-07

**Authors:** Marlene F. Schmidt, Zhong Yan Gan, David Komander, Grant Dewson

**Affiliations:** 1 The Walter and Eliza Hall Institute of Medical Research, 1G Royal Parade, Melbourne, VIC 3052 Australia; 2 Department of Medical Biology, University of Melbourne, Royal Parade, Melbourne, VIC 3052 Australia

**Keywords:** Macroautophagy, Ubiquitin ligases, Neural ageing, Neurological disorders

## Abstract

Neurodegenerative diseases are characterised by progressive damage to the nervous system including the selective loss of vulnerable populations of neurons leading to motor symptoms and cognitive decline. Despite millions of people being affected worldwide, there are still no drugs that block the neurodegenerative process to stop or slow disease progression. Neuronal death in these diseases is often linked to the misfolded proteins that aggregate within the brain (proteinopathies) as a result of disease-related gene mutations or abnormal protein homoeostasis. There are two major degradation pathways to rid a cell of unwanted or misfolded proteins to prevent their accumulation and to maintain the health of a cell: the ubiquitin–proteasome system and the autophagy–lysosomal pathway. Both of these degradative pathways depend on the modification of targets with ubiquitin. Aging is the primary risk factor of most neurodegenerative diseases including Alzheimer’s disease, Parkinson’s disease and amyotrophic lateral sclerosis. With aging there is a general reduction in proteasomal degradation and autophagy, and a consequent increase of potentially neurotoxic protein aggregates of β-amyloid, tau, α-synuclein, SOD1 and TDP-43. An often over-looked yet major component of these aggregates is ubiquitin, implicating these protein aggregates as either an adaptive response to toxic misfolded proteins or as evidence of dysregulated ubiquitin-mediated degradation driving toxic aggregation. In addition, non-degradative ubiquitin signalling is critical for homoeostatic mechanisms fundamental for neuronal function and survival, including mitochondrial homoeostasis, receptor trafficking and DNA damage responses, whilst also playing a role in inflammatory processes. This review will discuss the current understanding of the role of ubiquitin-dependent processes in the progressive loss of neurons and the emergence of ubiquitin signalling as a target for the development of much needed new drugs to treat neurodegenerative disease.

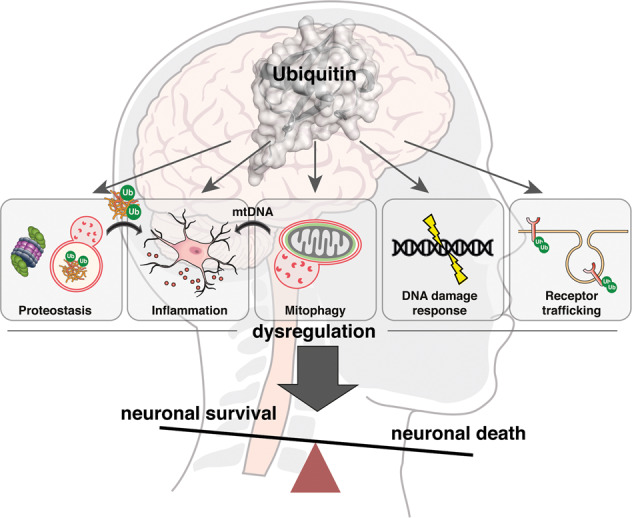

## Facts

- Genetic mutations and risk alleles found in neurodegenerative diseases including Alzheimer’s, Parkinson’s, Huntington’s and amyotrophic lateral sclerosis are associated with proteins involved in the ubiquitin–proteasome system (UPS) and the autophagy–lysosomal system.
- Ubiquitin and ubiquitin binding proteins are major constituents of the neurotoxic protein aggregates that characterise many neurodegenerative diseases.
- Dysregulated mitochondrial function supported by ubiquitin-mediated protein degradation pathways (UPS and mitophagy) are causally linked to neurodegenerative diseases.
- Non-degradative ubiquitin signalling is important for neuronal survival and function.
- Ubiquitin signalling is an emerging new target to diagnose and treat neurodegenerative conditions.

## Open questions

- Are disease-associated protein deposits an indicator of defective proteasomal degradation or a driver of proteasomal impairment, or both?
- Is aggregation the cause of neuronal toxicity or an adaptive strategy by which neurons (and other brain cells) sequester toxic proteins?
- Why are neurons selectively sensitive to defects in ubiquitin signalling?
- What is the interplay between different brain cell types in the pathogenesis of the neurodegenerative disease?
- Will targeting ubiquitin signalling limit neurodegeneration?

## Ubiquitin and proteostasis in neurodegenerative disease

The long-lived nature of neuronal cells and their inability to undergo division predisposes them to the toxic effects of accumulated misfolded proteins or damaged organelles. Neurons must therefore rely on quality control mechanisms, and the breakdown of these mechanisms is a hallmark of aging and negatively impacts neuronal health in neurodegenerative disease [1, 2]. Although most cases of neurodegenerative diseases are idiopathic with no clear genetic basis, some are caused by mutation in, or dysregulated expression of, genes that control protein turnover and degradation, including ubiquitin itself (Table 1) [3].

**Table 1:** Ubiquitin-signalling genes associated with neurodegenerative disease.

	Gene	Mutation, expression	Disease	Function/pathology	Ref
*Ubiquitin Ligases*	*CHIP*	Upregulated	AD	Several potential roles in AD, including the ubiquitination of phosphorylated tau.	[211]
	*HACE1*	Downregulated in striatum	HD	Implicated in the Nrf2-mediated antioxidative stress response.	[212]
	*HRD1*	Downregulated in cerebral cortex	AD	Regulates ER-associated degradation and is potentially involved in APP turnover.	[213]
	*Nedd4*	Upregulated	AD, HD, PD, ALS	Nedd4 family mediates endocytosis and lysosomal degradation of cell surface receptors (such as DAT, IGF-1R and AMPAR), regulates transmitter homoeostasis and synaptic plasticity.	[67, 69]
	*PRKN (PARK2)*	LOF mutation	PD	Parkin ubiquitinates outer mitochondrial membrane proteins to drive mitophagy. Also implicated in EGFR endocytic trafficking and inflammation.	[23]
	*RNF182*	Upregulated	AD	Regulates the turnover of an essential component of the neurotransmitter release machinery.	[214]
	*TRAF6*	Upregulated in *substantia nigra*	PD	Ubiquitinates α-synuclein and co-localises with Lewy bodies.	[215, 216]
	*TTC3*	Mutation, downregulated	AD	Regulates neuronal differentiation and Akt signalling.	[217, 218]
*DUBs*	*UCHL1*	Downregulated	AD, PD	Implicated in the degradation of disease-associated aggregates.	[178, 181]
		LOF mutation	PD		
	*USP9X*	Downregulated in *substantia nigra*	PD	Regulates α-synuclein ubiquitination and is present in Lewy bodies, regulates turnover of pro-survival MCL1.	[133]
	*USP9Y*	Downregulated (in males)	AD	Based on high sequence similarity with USP9X, may also regulate α-synuclein and phospho-tau.	[131]
*Autophagy receptors*	*OPTN*	LOF mutation	ALS	Major role in autophagy, including the removal of damaged mitochondria (mitophagy). Also implicated in other cell functions, such as innate immune response.	[22, 33]
	*p62/SQSTM1*	LOF mutation	ALS	Important for autophagy and aggrephagy and can be detected in intraneuronal inclusion bodies.	[219, 220]
*Other ubiquitin signalling genes*	*USP13*	Upregulated	AD, PD	Implicated in tau and α-synuclein clearance, as well as in Parkin ubiquitination.	[130, 132]
	*FBXO7*	LOF mutation	PD	A component of the SCF E3 ligase complex that is involved in PINK1/Parkin mitophagy.	[221]
	*LRSAM1*	LOF mutation	PD	Involved in receptor endocytosis and ubiquitinating intracellular bacteria for autophagic clearance.	[222, 223]
	*PINK1*	LOF mutation	PD	A Ser/Thr kinase responsible for ubiquitin and Parkin phosphorylation during mitophagy.	[26, 27]
	*TBK1*	LOF mutation	ALS	A multifunctional Ser/Thr kinase, phosphorylates IRF3 during innate immunity and phosphorylates autophagy receptors to enhancing binding to ubiquitin and LC3.	[224]
	*UBB*	Mutation	AD	Polyubiquitin precursor protein and a transcriptional +1 frameshift mutation that causes proteasome impairment and mitochondrial dysfunction.	[3]
	*UBQLN1*	Splice variants	AD	Promotes Lys63 polyubiquitination of APP to limit its trafficking and Aβ secretion	[76, 77]
	*UBQLN2*	LOF mutation	ALS, FTD	Shuttles ubiquitinated proteins, including protein aggregates, to the proteasome for degradation.	[10, 11]
	*VCP*	LOF mutation	ALS	An AAA-ATPase that extracts ubiquitinated proteins from membranes/structures, often for proteasomal degradation.	[225]

*LOF* Loss of function.

**Fig. 1:**
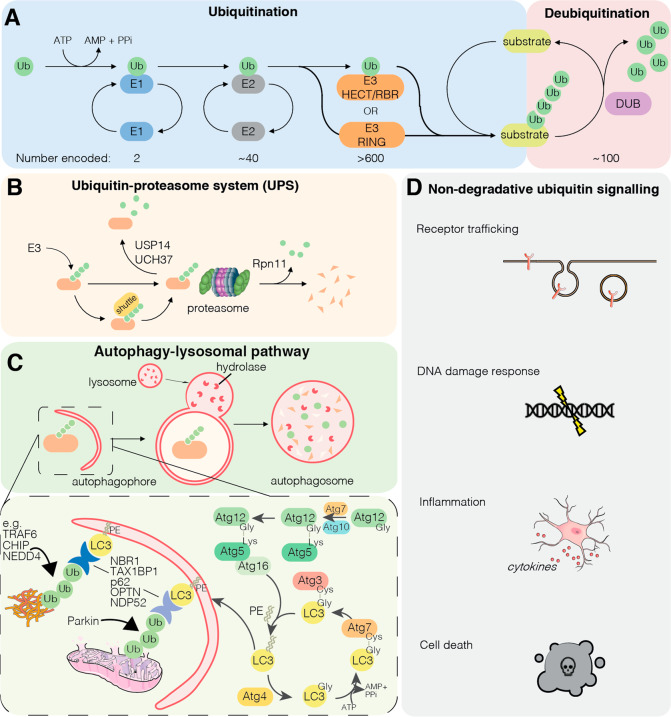
Ubiquitin in degradation and cell signalling. **A** *Schematic of the ubiquitination cascade and opposing deubiquitination*. During ubiquitination, ubiquitin is first attached to an E1 ubiquitin activating enzyme in an ATP-dependent manner before being transferred to an E2 ubiquitin conjugating enzyme. The Ub~E2 conjugate is then recognised by an E3 ubiquitin ligase, and ubiquitin is then transferred either directly to a substrate (for RING family E3 ubiquitin ligases) or first attached to the E3 ubiquitin ligase before being transferred to the substrate (for HECT and RBR family E3 ubiquitin ligases). The reverse reaction, deubiquitination, is catalysed by the deubiquitinating enzymes (DUBs) which remove ubiquitin from ubiquitinated substrates. **B** *Ubiquitin–proteasome system*. Ubiquitination of substrates can target proteins directly, or via ubiquitin-binding shuttle proteins such as UBQLN2, to the proteasome for proteolysis. Deubiquitination at the proteasome by proteasome-associated DUBs (USP14, UCH37) can rescue substrates from degradation. **C** *Autophagy–lysosomal pathway*. Specific E3 ligases ubiquitinate protein aggregates (e.g. CHIP, NEDD4, TRAF6), or damaged organelles (e.g. Parkin), to recruit autophagy receptors (e.g. p62, Neighbour of BRCA1 gene 1 (NBR1), OPTN, NDP52, TAX1BP1) that can simultaneously bind ubiquitin and Atg8-like proteins (LC3s). The Atg proteins catalyse the conjugation of the lipid phosphatidylethanolamine (PE) to LC3s and mediate the expansion of the autophagophore. **D** *Non-degradative ubiquitin signalling*. Ubiquitination can influence multiple pathways through degradative mechanisms or through non-degradative mechanisms. Non-proteolytic roles of ubiquitin often involve atypical chain linkage types.

Ubiquitin is a highly conserved 76 amino acid protein that is conjugated to substrate proteins through linkage via its C-terminal glycine residue. Modification typically occurs at the side chain of lysine residues or the N-terminal methionine, although recently serine, threonine and cysteine residues have also been identified as sites for ubiquitination [4]. The process of ubiquitination occurs through an enzymatic cascade involving the coordinated action of a hierarchy of increasingly specific and numerous enzymes: E1 ubiquitin-activating enzymes, E2 ubiquitin-conjugating enzymes and E3 ubiquitin ligases, and is counteracted by the deubiquitinases (DUBs) that detach ubiquitin molecules from substrates (Fig. 1A) [5].

Each of ubiquitin’s seven lysine residues, along with its N-terminal methionine, can be modified with ubiquitin to generate polyubiquitin chains of different linkage types. The nature of the linkage determines the fate of the modified protein. Although the myriad complexities of these linkages and their consequence are only starting to be resolved, the majority of ubiquitin signals direct tagged substrates for proteasomal degradation [5].

**Fig. 2:**
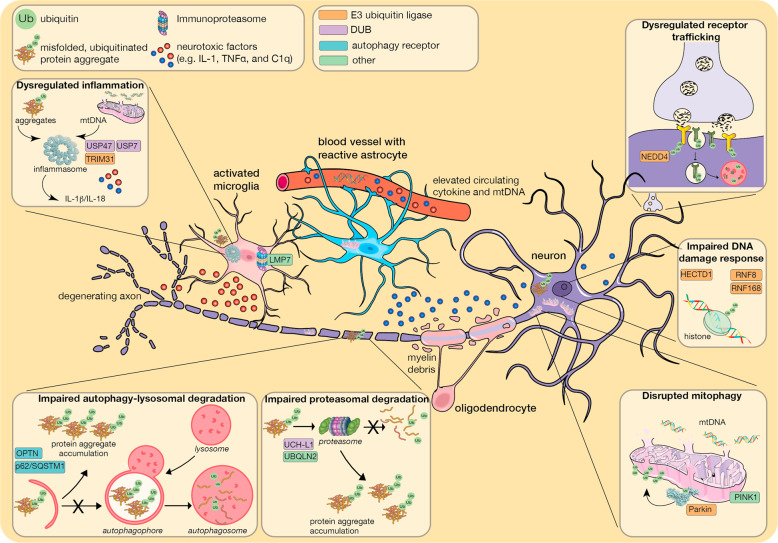
Ubiquitin signalling in the neurodegenerative brain. Degradation-dependent and degradation-independent ubiquitin signalling plays a fundamental role in neuronal functioning and survival and disrupted control of these processes can trigger neuronal loss. Defective clearance of misfolded and ubiquitinated protein aggregates (such as Aβ, tau in AD; α-synuclein in PD; htt in HD; SOD1, TDP-43 in ALS) due to *impaired proteasomal degradation* and/or *impaired autophagic–lysosomal degradation* are a hallmark of neurodegenerative disease. *Disrupted mitophagy:* The pathological effect of defective mitochondria is exacerbated by impaired ubiquitin-mediated mitophagy due to compromised PINK1 or the E3 ubiquitin ligase Parkin. Damaged mitochondria are a potent source of DAMPS (e.g. mtDNA) and damaging reactive oxygen species. *Dysregulated inflammation:* Toxic protein aggregates, mtDNA and myelin deposits trigger inflammasome formation and the release of pro-inflammatory cytokines such as IL-1β and IL-18 and other neurotoxic factors by reactive astrocytes and microglia. Increased activity of the immunoproteasome in microglia further drives an inflammatory response contributing to disease pathology. *Impaired DNA damage response*: Disrupted ubiquitin signalling in the DNA damage response (e.g. via perturbed ubiquitination of histones). *Dysregulated receptor trafficking:* Endolysosomal trafficking such as that mediated by NEDD4 regulates cell surface expression of key neuronal receptors including EGF and AMPA receptors.

A characteristic of many neurodegenerative diseases is misfolded protein aggregates in distinct regions in the brain, suggesting severe impairment in cellular protein degradation pathways [6, 7]. Typically, these aggregates consist of β-amyloid (Aβ) and hyperphosphorylated tau in Alzheimer’s disease (AD), α-synuclein in Parkinson’s disease (PD), huntingtin (htt) in Huntington’s disease (HD), and super oxide dismutase 1 (SOD1) or TAR DNA binding protein-43 (TDP-43) in amyotrophic lateral sclerosis (ALS). However, these aggregates, even Aβ plaques that form extracellularly, also commonly include ubiquitin and enzymes that catalyse its addition to substrates, further suggesting that defects in proteostasis underpin disease pathogenesis [8]. However, many types of non-degradative ubiquitin signals also play an essential role in signalling pathways (Fig. 1D) and are fundamental for neuronal functioning and survival including mitochondrial homoeostasis, membrane receptor trafficking and DNA damage responses (Fig. 2). Hence, defects in ubiquitin-regulated pathways are emerging as important drivers of neurodegenerative disease.

### The ubiquitin–proteasome system (UPS)

The main cellular mechanism for protein turnover is the UPS involving the 26S proteasome (Fig. 1B) [9]. Proteins modified with ubiquitin chains are commonly destined for proteasomal degradation as the attached ubiquitin chain is either recognised directly through the proteasome 19S regulatory particle [9] or is shuttled to the proteasome through association with ubiquitin-binding shuttle factors, including ubiquilin-2 (UBQLN2), a protein that is mutated in familial ALS and frontotemporal dementia (FTD) (Table 1) [10, 11]. Once bound at the proteasome, substrates are deubiquitinated by the DUB Rpn11 to recycle the ubiquitin, unfolded, and threaded into the proteolytic 20S core particle to be degraded [9]. The DUBs Ubiquitin-Specific Protease 14 (USP14) and Ubiquitin Carboxyl-terminal Hydrolase L5 (UCHL5/UCH37) associate with the proteasome to deubiquitinate incoming substrates to limit their degradation [9].

Proteasomal turnover represents a particular challenge for neurons due to their highly connected dendritic trees, long axons (e.g. motoneurons) and complex zones for presynaptic neurotransmitter release and postsynaptic receptor regulation [12, 13]. Hence, neurons are particularly sensitive to defects in proteasomal turnover and proteostasis. To overcome these challenges, neurons can regulate proteasomal activity and recruitment to distant dendritic spines in a synapse activity-dependent manner [14].

### Autophagy–lysosomal pathway

The Autophagy–lysosomal pathway involves ubiquitin-dependent degradation of cargo via lysosomes rather than the proteasome and is regulated by the coordinated activity of autophagy-related (*Atg*) genes (Fig. 1C). Autophagy is key for the degradation of cytoplasmic contents in response to cellular stresses such as starvation, but is also critical for the selective capture and degradation of particular cytoplasmic structures such as damaged mitochondria (*mitophagy*), invading microbes (*xenophagy*) and also protein aggregates (*aggrephagy*),

Various E3 ubiquitin ligases ubiquitinate specific cargo. For example, TNF Receptor-Associated Factor 6 (TRAF6), Neuronal Precursor cell Expressed Developmentally Downregulated (NEDD)4, and Carboxyl terminus of HSC70 Interacting Protein (CHIP) ubiquitinate specific aggregates of misfolded protein (reviewed by Le Guerroué and Youle [15]), whilst Parkin ubiquitinates outer membrane proteins on damaged mitochondria to mediate mitophagy (Fig. 1C). This ubiquitin platform recruits ubiquitin-binding autophagy receptors including p62/sequestosome 1 (SQSTM1), Optineurin (OPTN), Nuclear Domain 10 Protein 52 (NDP52) and Tax1 Binding Protein 1 (TAX1BP1) [16]. Often, autophagy receptors simultaneously bind to the ubiquitinated cargo via ubiquitin-binding domains, and to Atg8-like proteins: the GABA type A receptor-associated proteins (GABARAPs) and the microtubule-associated protein 1 light chain 3 (MAP1LC3s), that participate in autophagosome biogenesis [16]. In doing so, the autophagy receptors tether ubiquitinated cargo to the nascent phagophore, which subsequently fuses with lysosomes for cargo degradation and recycling (Fig. 1C).

Autophagy is a major player in removing misfolded aggregated proteins. This is highlighted by the accumulation of ubiquitin positive aggregates and neurodegeneration following the conditional deletion of the essential autophagy proteins Atg5 or Atg7 in neuronal cells [17, 18]. Disrupted autophagy is observed in AD and HD [19–21], and mutation of various autophagy-related genes drives the pathogenesis of various neurodegenerative diseases including PD and ALS (Table 1). For example, mutations in the autophagy receptor protein OPTN are linked to familial ALS [22], whilst loss-of-function mutations in proteins involved in the clearance of damaged mitochondria, including the E3 ubiquitin ligase Parkin, cause autosomal recessive juvenile onset forms of PD [23].

## Ubiquitin signalling in mitochondrial homoeostasis

Given that neurons are particularly reliant on mitochondrial oxidative phosphorylation to meet their significant energy requirements [24], it is not surprising that mitochondrial defects are emerging as important drivers of neurodegenerative diseases. Maintaining a healthy mitochondrial pool is particularly important given that mature, terminally differentiated neurons cannot employ cell division to dilute the impact of mitochondrial damage or dysfunction. To mitigate mitochondrial stress, numerous quality control mechanisms are employed, including a network of chaperones and proteases that maintain mitochondrial proteostasis and buffer against proteotoxic stress [25], and the selective degradation of damaged mitochondria via mitophagy.

### PINK1/Parkin-mediated mitophagy

**Fig. 3:**
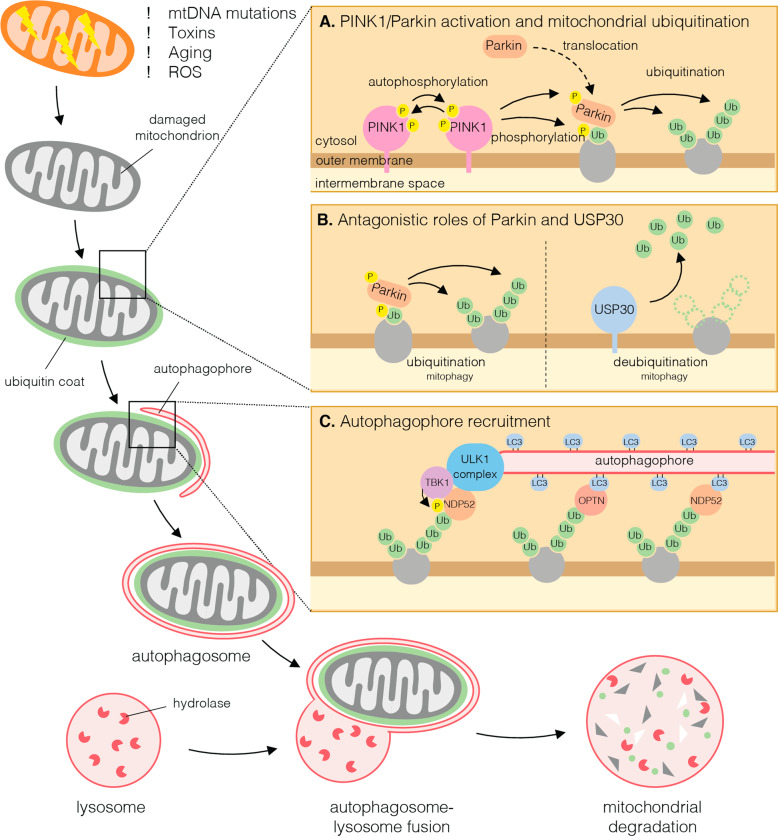
Parkin-mediated mitophagy. Chronic stresses of aging such as ROS and accumulated mitochondrial DNA (mtDNA) mutations provoke mitochondrial defects and damage that need to be counteracted by mitophagy to maintain neuronal cell survival. Upon mitochondrial damage, PINK1 is stabilised on the mitochondrial outer membrane where it autophosphorylates and activates (inset **A**). Active PINK1 phosphorylates ubiquitin, which recruits cytosolic Parkin to mitochondria. PINK1 then phosphorylates and activates phospho-ubiquitin-bound Parkin, enabling Parkin to ubiquitinate proteins on the mitochondrial outer membrane. Deubiquitinases, including USP30, remove ubiquitin from mitochondrial outer membrane proteins either upstream or downstream of Parkin activity to limit mitophagy (inset **B**). Mitochondrial ubiquitination leads to the recruitment of autophagy receptor proteins such as NDP52 and OPTN that is promoted by phosphorylation by TANK Binding Kinase 1 (TBK1), and the subsequent recruitment of the Unc-51 like autophagy activating kinase (ULK1) complex drives autophagophore biogenesis and encapsulation of mitochondria (inset **C**). Subsequent autophagosome-lysosome fusion leads to mitochondrial degradation by lysosomal hydrolases.

Mitophagy is intricately coordinated by multiple mechanisms in a context-dependent fashion. The most well characterised pathway is the ubiquitin-dependent clearance of damaged mitochondria regulated by the serine/threonine kinase PINK1 and the E3 ubiquitin ligase Parkin (*PRKN*/*PARK2*) (Fig. 3). This pathway is implicated in neurodegeneration since multiple loss-of-function mutations in either PINK1 or Parkin are known to cause autosomal recessive juvenile onset PD (Table 1) [23, 26].

PINK1 acts as a sensor of mitochondrial damage. In healthy mitochondria, PINK1 is rapidly imported into the mitochondria, cleaved, then degraded via the UPS [27]. However, upon mitochondrial damage, it is instead stabilised on the outer mitochondrial membrane where it dimerises and autoactivates [28, 29]. PINK1 then phosphorylates ubiquitin conjugated to mitochondrial outer membrane proteins, which recruits cytosolic Parkin. Parkin is then itself phosphorylated by PINK1 fully licensing its E3 ubiquitin ligase activity to ubiquitinate numerous mitochondrial proteins with mono and short chain ubiquitin signals, the latter featuring Lys6 and Lys11 linkages [30, 31]. This in turn provides more ubiquitin substrates for PINK1 to establish a positive feedback loop [32]. Ubiquitinated mitochondrial proteins recruit autophagy receptor proteins OPTN and NDP52, which facilitate autophagophore biogenesis and encapsulation of the mitochondria within the autophagosome [33, 34]. The autophagosome subsequently fuses with a lysosome to instigate mitochondrial degradation. This ubiquitin-dependent clearance of mitochondria is negatively regulated by DUBs including USP30 [35] and USP15 [36]. In opposing the activity of Parkin, USP30 preferentially cleaves Lys6 ubiquitin linkages that are catalysed by Parkin during mitophagy (Fig. 3) [37–39].

PINK1/Parkin-mediated mitophagy in response to mitochondrial damage can be induced in cultured cells and in vivo by mitochondrial toxins, some of which cause parkinsonism in humans [40, 41], and mitochondrial stress due to impaired mitochondrial protein homoeostasis and increased mutational load [42, 43]. However, the physiological trigger of mitochondrial damage in the brain of patients with familial early onset PD has not been resolved.

Early studies indicated that mice deficient for Parkin or PINK1 do not exhibit overt neuronal degeneration [44, 45]. However, a recent study revealed that *parkin* knockout mice do in fact show degeneration of dopaminergic neurons and consequent motor deficits when aged more than 2 years [46], supporting the compelling evidence that Parkin deficiency leads to parkinsonism in patients. Moreover, supporting the notion that Parkin responds to mitochondrial damage, dopaminergic neuron loss and motor deficits can be provoked in *parkin* knockout mice by mitochondrial stress due to accumulated mitochondrial DNA mutations caused by defects in the proofreading enzyme, DNA polymerase γ (PolG) [43].

Dopaminergic neurons are particularly susceptible to PINK1/Parkin deficiency in PD. However, Parkin loss may contribute to the pathogenesis of other neurodegenerative diseases as *parkin* mRNA expression is controlled by ALS-associated TDP-43 [47], tau impedes Parkin recruitment to mitochondria, and increased mitochondrial Parkin is observed in astrocytes of Apolipoprotein E4-carriers, a major genetic risk factor for AD [48–50]. Whether the pathogenic effect of Parkin loss and deficits in responding to mitochondrial damage is intrinsic to neurons in patients and animal models, or whether its loss of function in non-neuronal cells drives disease, is unclear.

## Ubiquitin signalling in the DNA damage response

Post-mitotic mature neurons have to deal with accumulated DNA damage as a consequence of chronic oxidative stress. DNA adducts and oxidative base damage are observed in nuclear and mtDNA in the aging brain as well as in neurodegenerative diseases, including AD, PD and ALS [51, 52]. E3 ubiquitin ligases play a critical role in base excision repair and determining cell survival in response to DNA damage. For example, DNA-damage Binding protein 1 (DDB1), through its interaction with the Cullin4 E3 ubiquitin ligase complex, and the E3 ligase HECTD1 mediate excision repair [53, 54]. Whilst an association with neurodegenerative disease in patients  has not been reported, DDB1 mutations are linked to the neurological disorder, Cockayne’s syndrome, and deletion of HECTD1 or DDB1 in mice promotes neuronal degeneration and neurodevelopmental defects [54, 55]. Intriguingly, DDB1 has also been shown to interact with Amyloid Precursor Protein (APP), suggesting a molecular link in AD [56].

The post-mitotic state of neurons reduces the risk of double strand DNA breaks, but also renders neurons particularly sensitive to their impact. The ability to repair such DNA damage declines with age and defects in the response are linked to neurodegeneration [57]. E3 ubiquitin ligases including RNF8, RNF168 and RAD18 are critical components of double-strand break repair by mediating histone 2A and 2B ubiquitination. A patient with homozygous mutation in the RNF168 ubiquitin binding domain exhibited mild ataxia-telangiectasia [58] and mice lacking RNF8 suffer from neuronal degeneration [59]. Furthermore, mice lacking RAD6B/UBE2B, the E2 ubiquitin-conjugating enzyme for the RING E3 ligase RAD18, have overt neurodegeneration due to a defective response to irradiation-induced DNA damage [60]. Toxic protein aggregates have been linked with DNA damage in neurodegenerative disease, with ectopic expression of mutant htt promoting extensive histone deubiquitination [61] and aggregates of p62/SQSTM1 in ALS and FTD able to inhibit the DNA repair ubiquitin ligase RNF168 [62].

If the DNA damage overwhelms the repair mechanisms a neuronal response is to trigger cell cycle re-entry and apoptosis. This control of cell cycle re-entry is potentially dysregulated in AD, as neurons from a transgenic AD mouse model, or isolated neurons treated with Aβ_1–42_ peptide, exhibited aberrant activation of the E3 ubiquitin ligase Itch, to provoke re-entry into the cell cycle due to Tap73 degradation, and consequently cell death [63]. Although the mechanisms and whether DNA damage is a driver or a consequence of neurodegenerative disease are still to be resolved, these data suggest that the ubiquitin-dependent response to DNA damage is an under-appreciated player in neurodegenerative diseases that can be compromised by protein aggregates.

## Ubiquitin signalling in endocytosis

Endocytic membrane trafficking of cell surface receptors is critical for neuronal survival and functioning, including synapse development and synapse plasticity [64]. As synapse loss and dysfunction is a key feature of neurodegeneration, defective endolysosomal trafficking is implicated in various neurodegenerative diseases including PD and ALS [65]. Receptor endocytosis and degradation through the endolysosomal pathway is governed by monoubiquitination or Lys63-polyubiquitination, with the extent of ubiquitination determining whether the receptor is targeted to the lysosome for degradation or trafficked back to the cell surface [66]. The NEDD4 family of Homologous to E6AP C-terminus (HECT) E3 ubiquitin ligases are upregulated in AD, PD and HD, and ubiquitinate various neuronal receptors to promote their lysosomal degradation, including the dopamine transporter that mediates uptake of dopamine into dopaminergic neurons, insulin growth factor-1 receptor that is important for neurotrophic signalling, and glutamate receptors that control synapse plasticity [67, 68]. In addition, Aβ-induced defects in synapse plasticity due to degradation of α-amino-3-hydroxy-5-methyl-4-isoxazolepropionate (AMPA) glutamate receptors involves ubiquitination by NEDD4-1 [69]. However, the link between NEDD4 and neurodegeneration is not straight forward as NEDD4-1 is also implicated in the endolysosomal degradation of α-synuclein in PD [70], suggesting its activity may be protective in certain conditions.

Akt signalling through the epidermal growth factor receptor (EGFR) provides survival cues to dopaminergic neurons. Parkin, in addition to its role in mitophagy, is implicated to inhibit clathrin-mediated endocytosis of EGFR through mono-ubiquitination of epidermal growth factor receptor substrate 15, and in doing so promotes Akt signalling and neuronal survival [71]. Additionally, Parkin mono-ubiquitinates the PDZ protein PICK1, which regulates trafficking of AMPA receptors thereby potentially regulating excitotoxicity [72, 73]. How Parkin is activated in these contexts, how this activity relates to its role in mitophagy, and the relative contributions of these seemingly distinct functions to the survival of dopaminergic neurons in PD is not resolved.

As well as regulating receptor signalling, endosomal trafficking can play an active role in neurodegeneration as extracellular protein aggregates can also be transmitted between neurons by endocytic uptake, thereby propagating the disease [74]. Endosomes are also the site for the processing of APP to aggregation-prone Aβ peptides. F-Box/Leucine rich repeat protein 2 (FBL2/FBXL2) is part of the SKP1-Cullin-F-box ubiquitin ligase complex that ubiquitinates APP to drive its degradation and also limit its entry into endosomes, hence Aβ plaques are reduced in FBL2-transgenic mice [75]. Likewise, UBQLN1, the mutation of which has been linked to AD, promotes APP ubiquitination to limit Aβ secretion [76, 77]. Together, these findings indicate that ubiquitin-mediated endolysosomal trafficking plays an important yet complex role in the progression of neurodegenerative disease.

## Ubiquitin signalling in glia and neuroinflammation

Although neurodegenerative diseases have long been considered neuron-autonomous disorders, an important role for non-neuronal glial cells (microglia, astrocytes, oligodendrocytes) in disease pathogenesis is emerging. Glial cells maintain brain homoeostasis and support neuronal survival and function [78]. However, in the degenerative brain, microglia and astrocytes display impaired phagocytotic clearance of unwanted material such as myelin debris, dead cells and protein aggregates [79], and adopt a reactive neurotoxic inflammatory phenotype [80]. Neuroinflammation is an established feature of neurodegenerative diseases including AD and PD, evidenced by elevated pro-inflammatory cytokine levels in the blood and cerebral spinal fluid [81] with mounting evidence suggesting that inflammation is a driver of disease pathology rather than a consequence.

mtDNA, myelin debris and disease-associated aggregates comprising Aβ, α-synuclein and tau, serve as Danger-Associated Molecular Patterns (DAMPs) that elicit a neuroinflammatory response. These DAMPs are sensed by pattern recognition receptors with protein aggregates detected by the inflammasome sensor protein, NOD-like receptor protein 3 (NLRP3) (reviewed in [82]). When activated, NLRP3, and other inflammasome sensors, form multi-protein complexes that act as platforms for the activation of caspase-1. Caspase-1 cleaves the pro-inflammatory cytokines Interleukin-1β (IL-1β) and IL-18 into their mature bioactive fragments and, in parallel, triggers the inflammatory cell death pathway of *pyroptosis*, which facilitates inflammatory cytokine egress (see below). Inflammasome assembly and activation is tightly controlled by ubiquitination [83]. Pre-clinical evidence suggests NLRP3, caspase-1 and IL-1β are promising therapeutic targets in several neurodegenerative diseases [84]. Compellingly, pharmacological inhibition of NLRP3 prevented α-synuclein pathology and dopaminergic neurodegeneration in PD mouse models [85], hence NLRP3 inhibitors are now entering clinical trials for the treatment of PD.

Consistent with the link between inflammatory signalling and neurodegeneration, a genome-wide association study revealed common genotypes between PD and autoimmune disease, including mutation of the E3 ubiquitin ligase TRIM31, which ubiquitinates NLRP3 to promote its degradation and so limit inflammasome activity [86, 87]. Conversely, deubiquitination of NLRP3 by USP7 and USP47 positively regulates inflammasome activation and USP47 is significantly downregulated in dopaminergic neurons from idiopathic PD patient post-mortem brain samples [84, 88].

Recently, an E3 ubiquitin ligase COP1/RFWD2 was revealed to control pro-inflammatory gene expression in microglia by promoting degradation of the transcription factor c/EBPβ [89]. Conditional deletion of COP1 in microglia triggered a pro-inflammatory response leading to complement C1q-dependent neurotoxicity that exacerbated tau-driven pathology in mice. Complement protein C3 is cleaved in human AD brain and is required for neurodegeneration in mouse models of amyloidosis and tauopathy [90], suggesting that glia-derived complement plays an important role in AD pathogenesis.

mtDNA released from damaged mitochondria ignites an inflammatory response via cGAS/STING or NLRP3 signalling [91–94]. Loss of Parkin, or its activator PINK1, promotes inflammatory signalling in response to mitochondrial stress, and strikingly, blocking inflammatory signalling was sufficient to rescue dopaminergic neuron loss in the *parkin*/*POLG* mouse model of PD [95]. Degeneration of dopaminergic neurons and motor deficits can likewise be induced in *parkin* knockout mice by triggering inflammation through the systemic administration of lipopolysaccharide (LPS) [96], further highlighting the role of Parkin in controlling inflammation. Although the respective roles of neurons and glia were not explored in these studies, *parkin* knockout mice do exhibit damaged mitochondria in mesencephalic glia [97], and Parkin deficiency in primary microglia exacerbated NLRP3 signalling upon LPS stimulation [98]. This suggests that disruptions to PINK1/Parkin-mediated mitophagy likely has an important role in controlling inflammatory signalling that is extrinsic to dopaminergic neurons. A recent study revealed that TDP-43 can provoke mtDNA release and cGAS/STING-mediated inflammatory responses in ALS models and patient samples [99], suggesting that mtDNA may act as a DAMP in diverse neurodegenerative diseases. As well as protecting the mitochondrial network and limiting mtDNA-driven inflammation, Parkin has also been shown to ubiquitinate RIPK1 to directly regulate inflammatory signalling through its control of nuclear factor-κB (NF-κB) [100, 101]. As in its proposed role in EGFR endocytosis, how Parkin is activated to target RIPK1 is not clear, but these data suggest that Parkin’s neuroprotective role may be multi-factorial.

Ubiquitinated protein aggregates are detectable in glia cells, potentially due to phagocytosis of neuronal elements or uptake of aggregates released from neurons [102]. However, glia are less vulnerable than neurons to their toxic effects, possibly due to higher UPS activity [103]. Furthermore, in addition to the 26S proteasome, microglia, as the predominant immune cell in the brain, also express the immunoproteasome, which processes peptides for antigen presentation and regulates inflammatory responses. Immunoproteasome subunits, such as LMP7, are upregulated in the brain of PD patients and α-synuclein aggregate-prone mice [104]. Likewise, in an AD mouse model and AD patient post-mortem tissue, Aβ plaques promoted immunoproteasome activity in proximal astrocytes and microglia, whilst inhibiting the immunoproteasome in microglia ex vivo decreased inflammatory signalling [105]. This raises the question whether the immunoproteasome might be more effective in degrading aggregated proteins. However, in the APP/PS1 mouse model of AD, although deficiency of LMP7 inhibited microglial cytokine response and improved cognition, it did not alter Aβ pathology [106].

Long thought of as a reaction to dying and dead neurons, dysregulated inflammation is now known to exacerbate, and possibly even trigger, the neuronal cell death that underpins disease pathology. As such, glial cells have come to the fore as therapeutic targets, highlighting the need for models where neuron:glia communication is retained.

## Ubiquitin signalling in neuronal cell death

Ultimately, the pathology of neurodegenerative diseases is due to the death of specific neuronal populations. However, it remains unclear why these neurons die as various triggers are implicated, including atypical protein aggregates, mitochondrial dysfunction, neuroinflammatory mediators or oxidative stress. In addition, *how* neurons die is also unclear, with different death modalities potentially playing a role.

*Apoptosis* is considered the main route to neuronal death in various neurodegenerative diseases [107], evidenced by DNA fragmentation and activity of the executioner caspase-3 in AD, PD and FTD patients [108–111]. X-linked Inhibitor of Apoptosis (XIAP) is an E3 ubiquitin ligase that ubiquitinates apoptotic caspases (including caspase-3, -7 and -9) to promote their degradation [112]. Nitric Oxide (NO), reported to inhibit XIAP’s ligase activity, is elevated in neurons with inflammation, and indeed, increased S-nitrosylated XIAP is found in brain tissue of patients with AD, HD and PD [113, 114], suggesting a potential role for XIAP in neurodegeneration. Other E3 ubiquitin ligases, including Parkin, are also S-nitrosylated in PD patient brains, although the data on the effect of NO on Parkin function and thus, a potential impact on cell death, are conflicting [115, 116].

Various ubiquitin-modulating enzymes regulate the intrinsic apoptotic machinery by targeting members of the BCL-2 family of proteins [117], and hence have been implicated in dictating neuronal survival and neurodegenerative diseases. Parkin can directly impair apoptosis by inhibiting the ability of the redundant BCL-2 effector proteins BAK and BAX to damage mitochondria, either by preventing mitochondrial localisation (BAX) or preventing oligomerisation (BAK) [118, 119]. Post-mitotic neurons are proposed to only express a truncated variant of BAK that lacks intrinsic apoptotic capacity, suggesting that BAX is likely key in apoptosis of mature neurons [120]. Consistent with this, BAX deletion is sufficient to protect neurons from death induced in vitro by the mitochondrial toxins 1-methyl-4-phenyl-1,2,3,6-tetrahydropyridine (MPTP) and 6-hydroxydopamine (6-OHDA) that promote parkinsonism [121, 122]. Conversely, inhibition of pro-survival protein MCL1 leads to BAX activation and dopaminergic neuron death [123] and deletion of *Mcl1* in *parkin* knockout mice promoted dopaminergic neuron death and motor deficits [124], suggesting that pro-survival BCL-2 proteins, and perhaps MCL1 in particular, are important to keep dopaminergic neurons alive in the face of mitochondrial defects. Various E3 ubiquitin ligases are reported to target MCL1 to promote its proteasomal turnover including HUWE1 [125] and also Parkin [126]. Whilst degradation of a pro-survival protein by the neuroprotective Parkin appears counter-intuitive, it might reflect a response to severe or prolonged mitochondrial damage [127]. Two DUBs USP9X and USP13 deubiquitinate and stabilise MCL1, and hypomorphic mutations in both have been linked to neurodevelopmental disorders and neurodegenerative disease [128, 129]. However, USP9X and USP13 target multiple substrates and are implicated in the clearance of disease-associated protein aggregates in AD [130, 131] and PD [132, 133], hence whether their deubiquitination of MCL1 is key to their neuroprotective function is unclear.

Apoptosis is largely considered a non-inflammatory mode of cell death. This potentially conflicts with neuroinflammation as a feature of certain neurodegenerative diseases and suggests that other more inflammatory modes of cell death may play a role in disease pathology.

*Necroptosis* is a caspase-independent, lytic form of cell death induced by a variety of stimuli, including ligation of cell surface death receptors such as TNF-α receptor 1 and Toll-like receptors (TLR). Necroptosis is executed by the oligomerisation of the pseudokinase Mixed Lineage Kinase-Like (MLKL) downstream of Receptor Interacting Protein Kinase 1 (RIPK1) and RIPK3, leading to the disruption of the plasma membrane and thus, to cell death and the release of pro-inflammatory stimuli and DAMPs [134–136]. RIPK1, RIPK3 and MLKL are activated in the brains of AD patients and their elevated expression correlated with disease progression [137, 138]. Moreover, motor deficits in an AD mouse model were partially rescued by pharmacological inhibition or genetic ablation of MLKL [137]. Necroptosis is also linked to PD, as phosphorylated MLKL (a marker of activation) was detected in brains of PD patients and of 6-OHDA-treated mice [139]. Deletion or inhibition of MLKL or RIPK1 protected neuronal cells, including human induced pluripotent stem cell-derived dopaminergic neurons, from degeneration driven by MPTP or 6-OHDA [140–142]. RIPK3 ablation likewise protected neuronal cells from MPTP-induced toxicity both in vitro and in vivo, although intriguingly this was due to impaired apoptosis rather than necroptosis [143]. A role for necroptosis signalling in ALS has also been proposed as pharmacological inhibition or genetic ablation of RIPK1 delayed symptom onset in the SOD1^G93A^ mouse model [144]. However, subsequent studies have questioned the role of necroptosis in ALS as neither a kinase dead mutant of RIPK1 [145], nor deletion of MLKL [146], delayed disease progression in the same mouse model.

Ubiquitination is a key checkpoint in death receptor signalling. Upon ligation of cell surface death receptors, RIPK1 is ubiquitinated by Cellular Inhibitor of Apoptosis (cIAP) 1 and cIAP2. This not only impairs its kinase activity, but also serves to recruit the linear ubiquitin assembly complex (LUBAC), and thereby diverts signalling from cell death to survival and pro-inflammatory signalling through NF-κB [147]. Interestingly, Parkin represents a molecular link between death receptor signalling and PD, as it is recruited to LUBAC to ubiquitinate the NF-κB Essential Mediator and promote cell survival signalling [148], whilst it also ubiquitinates RIPK1 to limit necroptosis signalling [101, 135].

*Pyroptosis*, like necroptosis, is a tightly regulated mode of inflammatory cell death. Inflammasome activation in response to various Pathogen-Associated Molecular Patterns and DAMPs, including aggregated α-synuclein and Aβ [149, 150], triggers activation of caspase-1 to cleave pro-inflammatory cytokines, but also the pore-forming protein gasdermin D, which permeabilises the plasma membrane leading to lytic cell death [151]. Whilst ubiquitin signalling regulates inflammasome activity in the initial phase of NLRP3 activation, whether ubiquitination controls the effector phase of pyroptosis is unknown. Although inflammasome signalling has been reported in neurons, consistent with its key role in innate immunity, pyroptosis is mainly described in microglia to potentially drive neuronal degeneration indirectly [152–154]. Caspase-1 inhibition reduced motor deficits in a 6-OHDA-induced rat model of PD [155], and also ameliorated the cognitive defects in a mouse model of AD [156]. NLRP3 and caspase-1 levels also correlated with neurons prone to degenerate in a mouse model of HD [157], and the NLRP3 inflammasome activation has been linked to FTD [158] and ALS [159]. However, NLRP3 activation in microglia in PD has been reported to occur in the absence of significant pyroptotic cell death [85], suggesting that cell death can be separated from IL-1β release. Therefore, it remains to be seen if pharmacological targeting of pyroptosis per se will be of benefit in these diseases.

*Ferroptosis* occurs as a result of excessive iron-dependent peroxidation of lipids [160]. Iron accumulation is a feature of the neurodegenerative brain and ferroptosis is implicated in PD, HD and AD (reviewed in [161]). Whilst relatively unexplored in the context of ferroptosis in neurons, ubiquitin signalling is emerging as a potential checkpoint. For example, in a non-neuronal setting, induction of ferroptosis with the small molecule erastin is suppressed by the ubiquitin ligase NEDD4 [162], whilst the DUBs OTUB1 and USP7 promote ferroptosis by stabilising the mediators SLC7A11 and p53, respectively [163, 164].

Understanding the pathway by which neurons die in specific neurodegenerative disease and how these pathways are controlled by ubiquitin signalling will undoubtedly lead to new opportunities for targeted intervention and the development of disease-modulating therapies that limit neuronal loss.

## Targeting ubiquitin signalling to treat neurodegenerative disease

**Fig. 4:**
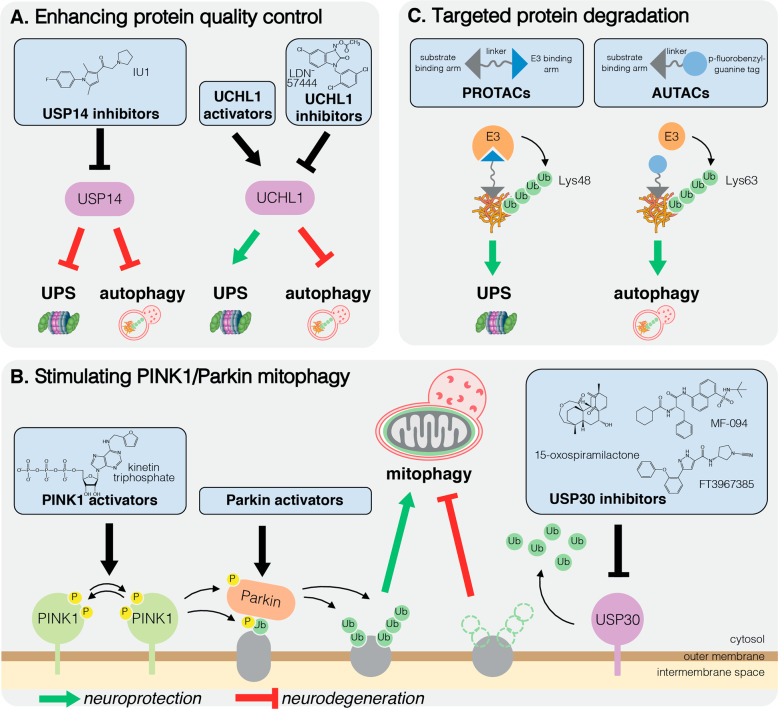
Ubiquitin signalling as a target to treat neurodegenerative disease. Defective ubiquitin signalling is an Achilles heel of neurons in neurodegenerative disease. Pre-clinical drug development to enhance neuroprotective (*green arrow*) or to inhibit neurodegenerative (*red block*) activity of targets, include stimulating protein quality control through modulating the deubiquitinating enzymes USP14 or UCHL1 (**A**), molecules that stimulate mitophagy by activating PINK1 or Parkin, or by inhibiting USP30 (**B**), and targeted degradation technologies (PROTACs and AUTACs) that recruit the ubiquitination machinery to induce selective ubiquitination and degradation of a target through either UPS or autophagy (**C**). Inhibitors of UCHL1 may also provide protection by stimulating aggrephagy. Compound structures were generated using ChemDraw 19.1.

There are currently no treatments that modify the underlying cause of neurodegeneration to halt or slow disease progression. There is a dire need for new therapies to combat the global rise in prevalence of neurodegenerative disease in an aging population. Whilst development of drugs targeting the ubiquitin system are in clinical trials as potential cancer therapies, in neurodegenerative disease such drugs are currently limited to preclinical studies. The diversity and specificity of the more than 600 E3 enzymes affords an attractive opportunity for precise targeting of disease-relevant pathways [165]. On the other hand, DUBs, of which there are ~100 encoded by the human genome, also represent promising targets to promote the degradation of neurotoxic aggregation-prone proteins [165] (Fig. 4).

### USP14 inhibition

USP14 is a DUB that has emerged over the last decade as a target to treat neurodegenerative disorders. USP14 is activated upon binding to the proteasome via its N-terminal ubiquitin-like domain and it removes ubiquitin chains to negatively regulate the degradation of select substrates [166]. Hence, USP14 knockdown enhances proteasomal degradation [167]. Notably, overexpression of USP14 in cultured cells spared ectopically expressed tau and TDP-43 from proteasomal degradation [168]. Conversely, a selective inhibitor of USP14, IU1, promoted degradation of overexpressed tau, TDP-43 and ataxin-3 [168] (Fig. 4A). Various derivatives of IU1 with increased potency have since been generated [169, 170], and were shown to be effective in enhancing the degradation of endogenous tau as well as exogenous mutant tau in primary neuronal cultures [169]. Although the effect of IU1 in animal models of neurodegenerative disease has not been tested, IU1 did reduce the severity of brain injury induced by ischaemia/reperfusion in mice [171], supporting its in vivo neuroprotective capacity.

Interestingly, it appears USP14 not only negatively regulates the UPS, but also negatively regulates autophagy through removing Lys63-linked ubiquitin chains from the autophagy regulator Beclin-1 [172]. Accordingly, pharmacological inhibition of USP14 enhanced autophagy and mitophagy in cultured cells [169, 172, 173], and USP14 knockdown partially reversed the locomotor deficits in PINK1/Parkin-deficient flies [173]. Thus, therapeutic inhibition of USP14 may promote protein degradation through UPS and autophagy pathways simultaneously.

Whilst USP14 is of interest as a target to treat neurodegeneration, there are certain issues that need to be resolved for it to progress as a clinical candidate. One study did not observe reduced degradation of tau and TDP-43 upon USP14 over-expression [174], while another study using rat neuronal cultures attributed the apparent IU1-induced tau degradation to cleavage of tau by calpain rather than the proteasome [175]. In addition, Kim et al. found that inhibition of USP14 decreased rather than increased autophagy and blocked autophagic clearance of mutant htt aggregates [176]. Moreover, as USP14-deficient mice show neurodevelopmental defects in synaptic transmission as well as neuromuscular defects [177], the potential clinical utility of USP14 inhibition to treat neurodegenerative diseases remains unclear [168].

### UCHL1 regulation

The ubiquitin hydrolase UCHL1 is implicated in the degradation of misfolded protein aggregates. Whilst (*UCHL1*/*PARK5)* is a PD susceptibility gene [178], its over-expression via intracranial administration of adenovirus also delays Aβ-induced neuronal loss in a mouse model of AD [179], suggesting that small molecule agonists may be broadly neuroprotective. However, somewhat surprisingly, small molecule inhibition of UCHL1 was actually found to limit α-synuclein aggregation in oligodendrocytes by stimulating autophagy [180] (Fig. 4A). Moreover, as pathogenic UCHL1 variants that have gained ubiquitin ligase activity have been identified in PD [181], like USP14, its utility as a target to treat neurodegenerative disease remains uncertain.

### Amplifying mitophagy to treat neurodegeneration

Given that hypomorphic mutations in Parkin or PINK1 cause early onset parkinsonism, there is particular interest in activating either protein to treat early onset disease [182]. However, as genetic inhibition of PINK1 enhanced α-synuclein aggregation in cultured cells [183] and exacerbated dopaminergic neuron loss in α-synuclein transgenic mice [184], stimulating PINK1/Parkin-mediated mitophagy could also be protective in sporadic disease (Fig. 4B). Additionally, mitochondrial dysfunction is a common feature of neurodegenerative disease, hence amplifying PINK1/Parkin mitophagy may also be beneficial in a broad spectrum of diseases including AD and HD [185, 186].

### Parkin activation

The structural and mechanistic details of Parkin activation have been resolved over the last decade [187–193]. As a Ring-Between-RING E3 ligase, Parkin comprises multiple domains, including the RING1 and RING2 domains, the IBR (In-Between-RING) domain, the UPD (Unique Parkin Domain; also known as RING0) and an N-terminal ubiquitin-like (Ubl) domain [194]. Parkin is autoinhibited under basal conditions as the catalytic Cys431 in the RING2 that receives a ubiquitin molecule during catalysis is buried in a hydrophobic interface formed between the RING2 and the UPD [187–189]. Parkin is activated in a stepwise fashion. First, the binding of phospho-ubiquitin leads to the release of the Ubl domain from the Parkin core [190, 193]. Secondly, phosphorylation of the Ubl domain by PINK1 enables the phospho-Ubl to bind the UPD, thereby dislodging the RING2 domain from its bound state and exposing the catalytic Cys431 to receive a ubiquitin from a charged E2~ubiquitin conjugate [191, 192]. The intricate understanding of this highly coordinated reconfiguration may enable the development of small molecules that target specific functional domains of Parkin to trigger discrete steps in its activation pathway [182, 191, 195]. Targeting intermediate conformers of Parkin rather than the autoinhibited form may limit on-target activity in non-target tissues such as heart, whilst also avoiding the potential off-target ubiquitination of cytosolic proteins, including Parkin itself.

### PINK1 activation

Compared with Parkin, opportunities to activate PINK1 are less well defined as the structure of human PINK1 has not yet been solved. However, given that PINK1 autophosphorylation in *trans* is required for PINK1 kinase activity [28, 29], promoting dimerisation and/or autophosphorylation are potential therapeutic strategies. Despite our incomplete understanding of PINK1 at the molecular level, efforts have been made to target and activate PINK1 using the ATP analogue kinetin triphosphate (KTP) [196, 197] (Fig. 4B). Compared with ATP, KTP enhanced activity of recombinant PINK1 and PINK1 in cells, demonstrating its therapeutic potential [196]. Indeed, KTP treatment restored locomotor activity and aberrant mitochondrial morphology in PINK1 knockdown flies [198]. Interestingly, the precursor of KTP, kinetin (a plant cytokinin), is metabolised to KTP when added to cells [196], but whilst long-term oral administration of kinetin was tolerated in rats, it failed to prevent neurodegeneration in response to α-synuclein aggregates [199]. However, it will be important to test whether kinetin treatment can limit neurodegeneration in models of PD involving mitochondrial stress or impaired mitophagy.

### USP30 inhibition

As opposed to activating PINK1/Parkin, another strategy to amplify mitophagy would be to inhibit negative regulators. Several candidates have been considered in this regard, with USP30 currently the most well-studied. USP30 is a mitochondria-anchored DUB that preferentially hydrolyses Lys6-linked ubiquitin chains on mitochondrial substrates shared with Parkin to set a threshold for mitophagy [35, 37–39, 200]. In a key study, Bingol et al. showed that USP30 overexpression limited PINK1/Parkin-mediated mitophagy in cells. Conversely, USP30 knockdown enhanced mitophagy in rat neuronal cultures and in vivo in PINK1- or Parkin-deficient flies [35].

These mechanistic studies demonstrated the therapeutic potential of small molecule USP30 inhibitors, the first of which, 15-oxospiramilactone, was identified from a phenotypic screen for compounds that rescue mitochondrial morphology defects in mitofusin1-deficient cells [201] (Fig. 4B). Kluge et al. reported the phenylalanine derivative MF-094 as a selective inhibitor of USP30, which accelerated mitophagy in C2C12 myotubes [202]. Recently, Rusilowicz-Jones et al. reported a N-cyano pyrrolidine derivative (FT3967385) inhibited USP30 with an IC_50_ of 1.5 nM and, whilst it showed some off-target inhibition of USP6, enhanced mitophagy in cultured neuroblastoma cells [200] (Fig. 4B). USP30 governs mitochondrial protein import at the Translocase of the Outer Membrane complex [203], suggesting that USP30 inhibition could have potentially toxic effects. That *Usp30* knockout mice are viable with no gross developmental phenotype raises confidence that USP30 could be targeted pharmacologically [203], however, studies in aged (and challenged) *Usp30* knockout mice will be imperative to understand the long-term consequence of USP30 inhibition.

### Hijacking ubiquitin signalling to treat neurodegenerative disease

Whilst elements of ubiquitin signalling are targets in their own right, hijacking ubiquitin-dependent degradation is also emerging as a potential therapeutic avenue to treat neurodegeneration. Targeted protein degradation technologies have emerged over the last decade as a novel strategy to clear neurotoxic proteins from cells. This technology relies on a heterobifunctional peptide or small molecule that simultaneously binds a protein of interest and a component of the UPS or autophagy machinery, thereby directing the protein towards degradation (Fig. 4C). The most well-established are the *proteolysis targeting chimeras* (PROTACs), which bind an E3 ubiquitin ligase to induce Lys48 polyubiquitination and proteasomal degradation of a target protein. Peptide-based PROTACs that degrade tau and α-synuclein aggregates have been developed [204–206]. The tau-degrading PROTACs fused a tau-binding motif from β-tubulin to peptides that bind VHL or KEAP1, adaptor proteins for Cullin E3 ligase complexes [204, 205]. The α-synuclein PROTAC fused an α-synuclein-binding peptide from β-synuclein to a C-terminal RRRG degron to recruit E3 ubiquitin ligases [206]. Both were conjugated to a cell penetrating peptide to promote cellular uptake [204–206]. Chu et al. further showed that their PROTAC could promote tau degradation in vivo in an AD mouse model, although whether it impacted cognition was not assessed [204].

Compared to peptide PROTACs, small molecule PROTACs are likely to have better bioavailability and blood-brain barrier permeability. They can also be designed to distinguish neurotoxic misfolded proteins from the physiological form, thus retaining the physiological function of the protein. Tomoshige and colleagues developed a small molecule PROTAC that exploited the E3 ubiquitin ligase cIAP1 to degrade htt in HD patient-derived fibroblasts [207, 208]. Based on a tau-binding positron emission tomography tracer, a small molecule PROTAC, QC-01-175, could recruit the E3 ligase substrate receptor Cereblon to specifically degrade disease-associated tau thereby limiting the sensitivity of stressed neurons derived from FTD patients [209].

Targeted degradation technologies exploiting autophagy have also been developed, including *autophagy targeting chimeras* (AUTACs). AUTACs incorporate a guanine-derived *p*-fluorobenzylguanine tag to induce Lys63-linked ubiquitination and autophagic degradation of target proteins, and also organelles [210]. However, as the UPS and autophagy machineries are often compromised in neurodegenerative disease, the concept of exploiting these machineries as a therapeutic strategy to remove disease-associated aggregates, or dysfunctional organelles such as mitochondria, has not been fully validated.

Chronic neurodegenerative diseases develop over decades. As neurons are terminally differentiated, the neuronal loss in these diseases is usually irreversible. Importantly, there are no treatments that stall neuronal loss to slow or stop disease progression and current therapies are limited to symptomatic treatments. Given the key role for ubiquitin signalling in many facets of neuronal function and survival, targeting ubiquitin signalling either to enhance the clearance of toxic protein aggregates or to rescue specific vulnerabilities as drivers of neuronal degeneration may represent game-changing treatments that slow or even stop neurodegenerative disease progression. Furthermore, such pharmacological intervention may augment exciting yet challenging stem cell transplantation or glia-neuron transdifferentiation strategies that may actually reverse the disease.
